# Endoreplication in the *Drosophila melanogaster* prothoracic gland is dispensable for the critical weight checkpoint

**DOI:** 10.17912/micropub.biology.000741

**Published:** 2023-02-21

**Authors:** MaryJane Shimell, Michael B O'Connor

**Affiliations:** 1 Genetics, Cell Biology and Development, University of Minnesota, Minneapolis, Minnesota, United States

## Abstract

Critical weight (CW) attainment is a key life event in the development of holometabolous insects including *Drosophila melanogaster.* It indicates that sufficient growth has occurred to initiate the juvenile-to-adult transition. The prothoracic gland (PG), the major insect larval endocrine organ, is a polyploid tissue that plays a key role in the determination of CW via release of the steroid hormone ecdysone. Here we show that when the cells of the PG fail to make the mitotic-to-endocycle switch, but instead remain mitotic, the result is more but smaller cells. Nevertheless, they reach the same CW and produce healthy adults after only a moderate developmental delay. We propose that the CW checkpoint can be reached by either an endocycling or mitotic PG and may simply reflect the attainment of sufficient ecdysone biosynthetic capacity to initiate metamorphosis.

**Figure 1. Developmental time and CW of endocycling and mitotic PGs in Drosophila larvae are similar:**
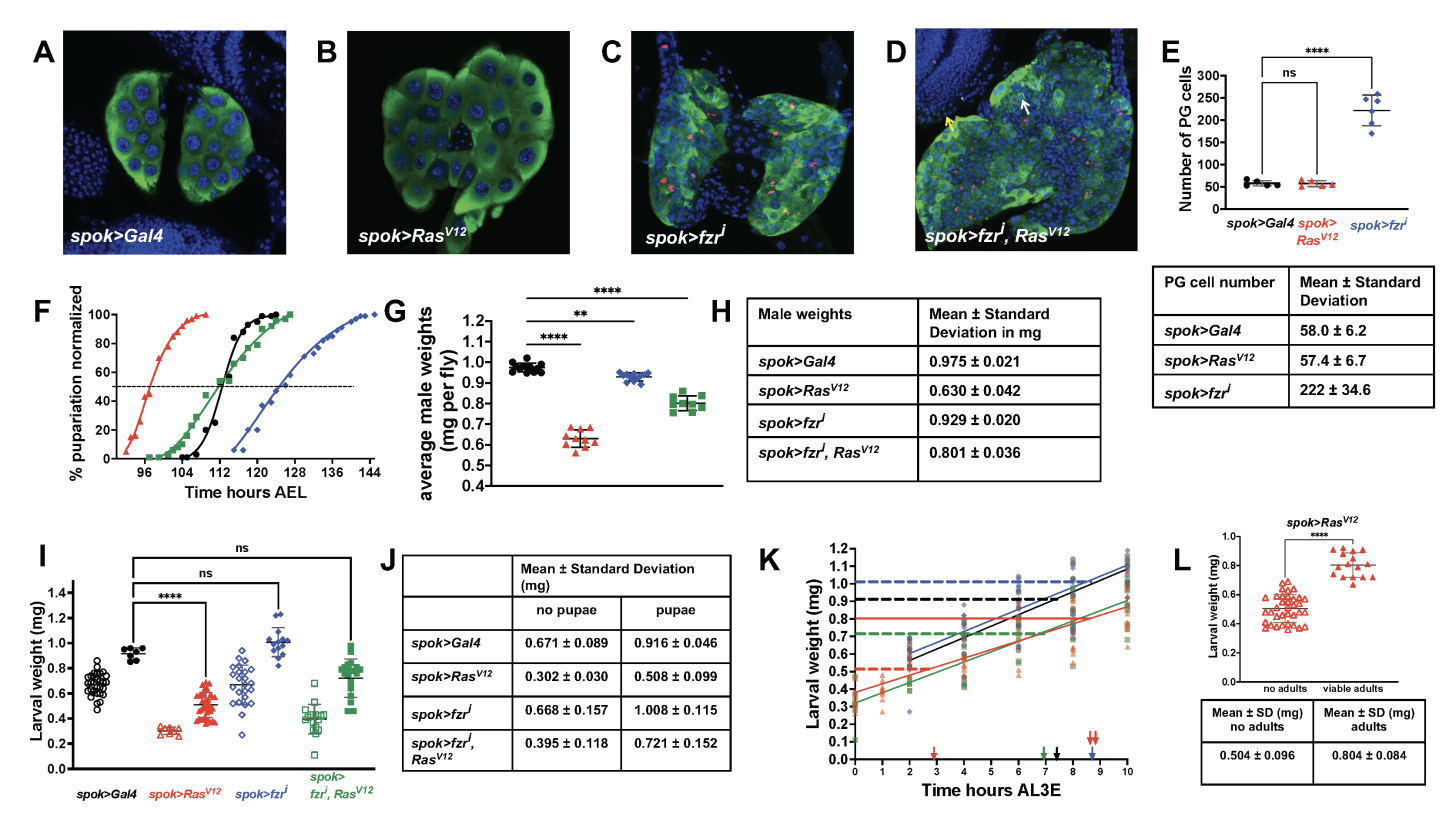
In all illustrations, the symbol for *spok>Gal4* is a black circle, *spok>fzr ^i^* is a blue diamond, *spok>Ras ^V12^* is a red triangle, and *spok>fzr ^i^ , Ras ^V12^* is a green square. A-D. Immunohistochemistry of wandering third instar larval prothoracic glands. Green staining is anti-Spok antibody, red staining is anti-Phospho-histone 3 (PH3) antibody, and blue staining is DAPI. Note the smaller cell size, increased cell number, and anti-PH3 staining when *fzr* is knocked down (Compare A to C and D). Note the increased cell size, nuclear size, and overall PG size when *Ras ^V12^* is expressed in the PG (Compare A to B). In *spok>fzr ^i^* , *Ras ^V12^* , compare the nuclear size of a PG cell (D, white arrow) to a mitotic brain nucleus (D, yellow arrow). E. Number of PG cells at CW and descriptive statistics. Above, serial z-stack sections were used to quantify the number of cells by counting the nuclei. Ordinary one-way ANOVA using Dunnett’s multiple comparison test (Prism) was used to show that cell numbers for *spok>Gal4* are statistically the same as *spok>Ras ^V12^* and about 4-fold higher for *spok>fzr ^i^* . ****P<0.0001. Below, descriptive statistics of PG cell number. F. Developmental timing curves. Nonlinear regression using Prism software with 50% pupariation indicated by the black dashed line. G. Adult male weights. Two vials of each cross yielded 9-11 batches of 6-10 males/batch. Each batch was individually weighed, and that weight/fly is shown. Note the decreased size of adults in all comparisons to *spok>Gal4* . Ordinary one-way ANOVA statistics using Dunnett’s multiple comparisons test (Prism). **** P<0.0001 **P=0.0015. H. Descriptive statistics of adult male weights. I. Critical weight determination. Empty symbols are larval weights at the start of starvation that did not yield pupae. Filled symbols are larval weights that did give rise to pupae. Kruskal-Wallis test of multiple comparisons to *spok>Gal4* (Prism) larval weights that yielded pupae. ****P<0.0001, P>0.9999 for *spok>fzr ^i^* , P=0.1643 for *spok>fzr ^i^ , Ras ^V12^* . J. Descriptive statistics of larvae that did or did not give rise to pupae. K. Larval weight gain between L3 ecdysis and 10 hours after L3 ecdysis (AL3E). Larval weights measured at the end of the feeding period. Nonlinear regression results in the straight lines (least squares fit, Prism) with the formulas: 0.92=0.61x + 0.45 for *spok>Gal4* , 0.51=0.49x + 0.38 for *spok>Ras ^V12^* , 1.01=0.6x + 0.47 for *spok>fzr ^i^* and 0.72=0.58x + 0.32 for *spok>fzr ^i^ , Ras ^V12^* . The CW (y in the formulas) is shown as a dashed horizontal line. The formulas were then used to interpolate the time at which CW occurred, pictured as arrows on the X axis. The interpolated time that MVW occurred in *spok>Ras ^V12^* is shown as a double red arrow on the X axis. L. Minimum viable weight determination and descriptive statistics. Above, empty triangles are larval weights of *spok>Ras ^V12^* at starvation initiation which gave rise to pupae but no adults. Filled triangles are larval weights that gave rise to pupae as well as adults. Welch’s t test of data (Prism) with descriptive statistics below. ****P<0.0001.

## Description

Endoreplication of the genome is a key property of various tissues and is often important for proper development of many plant and animal species. During endoreplication, multiple rounds of DNA synthesis (S) and gap (G) phases take place without cell division (mitosis, M phase), resulting in a tissue, or part of a tissue, that is polyploid. Most tissues in the *Drosophila melanogaster* larva grow through endoreplication, the most famous of which is the larval salivary gland where 9 rounds of reduplication lead to chromosomes that reach 1024 C values (Swift, 1962 and Nordman et al. 2011). These salivary gland polytene chromosomes were exploited historically to cytologically locate genes and regions of transcription using bands, puffs and other landmarks (reviewed in Zhimulev et al. 2004). Ploidy lends an advantage to a tissue since DNA is amplified (and hence the potential for high transcription levels of specific genes) without expending the energy or resources needed for cell division. One of the ways the mitotic-to-endocycle switch (MES) can occur is by inhibition of cyclin-dependent kinases (CDKs) via the Fizzy related (Fzr/Cdh1) subunit of the anaphase-promoting complex/cyclosome (APC/C) (see Fox and Duronio, 2013 and Edgar et al. 2014 for reviews). In the *Drosophila* prothoracic gland (PG), MES occurs between the first and second larval instars and subsequent endocycles continue through to the end of the third instar stage until a C value of 64 is reached at pupariation (Ohhara et al. 2017). Polyploidy in the PG is thought to aid in rapidly ramping up synthesis of the ecdysone biosynthetic enzymes leading to the peak of ecdysone required to initiate pupariation and metamorphosis.

In holometabolous insects, a key nutritional checkpoint that must be satisfied for the juvenile-to-adult transition to occur properly is referred to as Critical Weight or size (CW) (Beadle et al. 1938). In *Drosophila* , CW is operationally defined as the larval weight at which starvation will no longer delay or prevent pupariation. CW is invariant for a given genotype, but the time it takes to achieve it is dependent on diet, the rate of weight gain, and the environment. In *Drosophila* , starvation prior to CW results in no puparium formation or very delayed pupariation, while starvation after critical weight shortens the time to pupariation compared to non-starved controls (Beadle et al. 1938). Functionally, the CW checkpoint is thought to help ensure that larvae have sufficient energy stores to fuel metamorphosis during the non-feeding pupal stage. It has been well established that the PG plays a key role in mediating the CW response and much work has focused on determining how *Drosophila* detects when the CW checkpoint is reached *.* Manipulations of the environment, such as hypoxia (Callier et al. 2013), increased temperature (Ghosh et al. 2013), or low nutrition (Vijendravarma et al. 2012) decrease CW. Loss of the neuropeptides Prothoracicotropic hormone (PTTH), Jelly belly (Jeb) and PDGF- and VEGF-related (Pvf) as well as their Receptor Tyrosine Kinase Receptors Torso, Anaplastic lymphoma kinase (Alk), PDGF- and VEGF-related factor receptor (Pvr) and downstream effectors (Ras/Erk) increase CW and the time it takes to reach the checkpoint (McBrayer et al. 2007; Rewitz et al. 2009; Shimell et al. 2018; Pan and O’Connor, 2021). Similarly, overexpression of Forkhead Box class O (FoxO) in the PG also increases CW (Koyama et al. 2014), while suppression of autophagy pre-CW interferes with CW assessment (Pan et al. 2019) as does blockage of lipid transport from the fatbody to the PG (Juarez-Carreño et al. 2021). In a recent study, Ohhara et al. (2017) offered an intriguing potential molecular explanation for how CW might be determined via a counting mechanism. These authors noted a correlation between endoreplication cycle number and CW. They found that knockdown of *fzr* in the PG leads to continued mitotic divisions and developmental arrest in the third instar larva prior to CW. They reasoned, since ecdysone biosynthetic gene expression as well as ecdysone titers were low and feeding 20-hydroxyecdysone (20E) could rescue pupariation, that endocycles were necessary for attainment of CW.

In an effort to derive mitotic PG cells for possible cell culture, we expressed a combination of *fzr* RNAi (to maintain mitosis) together with a constitutively active form of Ras85D (Simcox et al. 2008) in the PG. For these experiments, we decided to use the *spookier (spok)* enhancer-promoter since it expresses specifically in the PG (Ono et al. 2006), whereas the *phm ^22^ -Gal4* line (Ono et al. 2006) used by Ohhara et al. (2017) and many others, expresses in several additional tissues such as trachea, enocytes, wing disc and select muscles (Mansilla et al. 2016 and our own observations). Since the available *spok>Gal4* lines were deemed “weaker” than *phm ^22^ -Gal4* , we generated numerous new random individual P element-mediated *spok-Gal4* insertions, as well as several recombinant two copy lines *,* and then examined them for PG-specificity and strength. We found that expression of the recombinant line *spok-Gal4-16A3,17A3* was not only PG specific, but was also at least equal in strength to *phm ^22^ -Gal4* as determined by GFP fluorescence in the PG upon crossing with a *UAS-GFP* reporter line. We also did a comparative phenotypic analysis using several RNAi lines known to affect ecdysone production including *dib* , *phm* , *spok,* and *smox* and found that the *spok-Gal4-16A3,17A3* line produced largely equivalent phenotypes to that produced by *phm ^22^ -Gal4* . Therefore, it was surprising when *spok-Gal4-16A3,17A3* (hereafter called *spok>* ) was crossed to *UAS-RNAi fzr* (hereafter called *fzr ^i^* ), either alone or in combination with constitutively active RasD85 *(Ras ^V12^ ),* we found that the resulting progeny were viable.

Given the dramatic difference in outcomes concerning how *fzr* RNAi knockdown in the PG affected the larval-adult transition in our experiments compared to those reported by Ohhara et al. (2017), we decided to reexamine the PG endocycle-critical weight connection. The central question we addressed first was whether *spok> fzr ^i^* eliminates endocycling as reported by Ohhara et al. using the *phm ^22^ -Gal4* driver. As shown in Figure 1, *spok> fzr ^i^* leads to a dramatic increase in PG cell number at the third instar wandering stage compared to the *spok>* control line (compare Figure 1A to Figure 1C and see Figure 1E for numbers at CW). Furthermore, these cells are much smaller on average than control PG cells and many are still mitotic as indicated by sporadic phospho-histone 3 positive cells in the *spok> fzr ^i^* but not the *spok>* control line (Figure 1A, 1C and 1D). We also examined the effect of overexpression of *Ras ^V12^* alone and the *fzr ^i^ , Ras ^V12^* combination on PG cell number and size. As shown in Figure 1B vs A, overexpression of *Ras ^V12^* enhances the cell size but does not appear to affect cell number compared to the *spok>* control consistent with previous observations (Caldwell et al. 2005 and Figure 1E). In the combination line, however, we once again observe a dramatically increased number of smaller and mitotically active cells compared to *Ras ^V12^* overexpressionalone (Figure 1B and 1D). These results indicate that *spok>* driven *fzr ^i^* knockdown is sufficiently strong to produce mitotically cycling cells, yet these larvae appear healthy and produce viable adults.

It is important to note, however, that while the nuclear size of *fzr ^i^* knockdown PG cells is significantly smaller than endoreplicating PG cells, it is larger than in mitotic diploid brain neurons or imaginal disc cells that appear in the same image plane (Figure 1D arrows for neurons vs *fzr ^i^* PG nuclear size comparison). Therefore, assuming similar nuclear packing, it may be that *fzr ^i^* knockdown alone does not produce a typical cell cycle. For example, these cells may have a larger C value of 4N and 8N at G1 and G2, respectively, similar to tetraploid cycling cells. In addition, we note that there are occasional rare nuclei which are significantly larger than others within the *fzr ^i^* knockdown gland reminiscent of true endocycling cells. Once again this might indicate that either *fzr ^i^* knockdown is not complete, or that to achieve true mitotic cycles, additional processes must be manipulated.

To further determine if PG cell size and endocycling has any more subtle developmental effects besides an absence of lethality, we examined the time to pupation, determined the CW, and quantified adult body size of our various *spok> Ras ^V12^* and *fzr ^i^* genetic combinations. The results of the developmental timing assay (Figure 1F) established that, relative to the *spok>* control (50% pupariation at 112 hours after egg lay (AEL)), *spok>fzr ^i^* is 15 hours delayed (50% pupariation at 127 hours AEL), whereas *spok>Ras ^V12^* is 14 hours accelerated in its timing (50% pupariation at 98 hours AEL). The *spok> fzr ^i^ , Ras ^V12^* pupariation profile was spread over a longer period but reached 50% pupariation at about the same time as the *spok>* control (Figure 1F). Even though *spok>fzr ^i^* flies took moderately longer to develop, the adults are significantly smaller than *spok>* (Figure 1G and 1H), which suggests that *fzr ^i^* knockdown in the PG leads to a slower growth rate (mass accumulation) than the control and that this slow growth rate is not fully compensated by the increased developmental time. In contrast, *spok>Ras ^V12^* adults are also significantly smaller than *spok>* controls *,* but they exhibit notably accelerated timing. Therefore, accelerated timing is not compensated by an increased growth rate. Lastly, *spok> fzr ^i^ ,Ras ^V12^* adults are intermediate in size to *spok>Ras ^V12^* and *spok>fzr ^i^* (Figure 1G and 1H) but remain significantly smaller than *spok>* flies. Taken together these results suggest that the growth rate-developmental timing relationship is altered in these genetic backgrounds compared to controls.

Since the *fzr ^i^* knockdown animals survive to adults, one might predict that these larvae are able to reach CW, despite having a mitotic PG. To examine this issue, we determined the CWs of *spok>, spok>fzr ^i^ , spok> fzr ^i^ ,Ras ^V12^* and *spok>Ras ^V12^* larvae *.* We found no significant difference among the critical weights for *spok>* (0.92 mg at 8 hours feeding after L3 ecdysis (AL3E) very similar to our previous determination of 0.86 mg (Shimell et al. 2018)), *spok>fzr ^i^* (1.01 mg at 9 hours AL3E), and *spok> fzr ^i^ ,Ras ^V12^* (0.72 mg at 7 hours AL3E) (Figures 1I, 1J, 1K), leading us to conclude that a mitotic PG can reach a CW that is not statistically different from the CW of an endoreplicating PG. In contrast to these genotypes, *spok>Ras ^V12^* larvae reach a critical weight of 0.51 mg which, under our growth conditions, occurs only 3 hours AL3E (Figs 1I, 1J, 1K). This is a statistically significant decrease in CW compared to any of the other lines. It is also interesting to note that only *spok>Ras ^V12^* larvae which weigh 0.80 mg prior to starvation (10 hours feeding of *spok>Ras ^V12^* ) gave rise to pupae that eclosed adults (Figures 1K and 1L). This leads us to conclude that in *spok>Ras ^V12^* larvae the CW differs from the minimum viable weight (MVW) even though it is generally accepted (Bakker, 1959 ; Mirth et al. 2005) that in wild type *Drosophila melanogaster* CW is the same as MVW since the majority of puparia that form in the critical weight assay give rise to adults, just as we observed with *spok>, spok>fzr ^i^ ,* and *spok> fzr ^i^ ,Ras ^V12^* lines. We know of only two other examples where CW and MVW can be separated in *Drosophila;* one involves manipulating insulin signaling in the PG (Mirth et al. 2005) and the other is observed in *ptth* null mutants (Shimell et al. 2018), which interestingly are also disrupted in Ras signaling.

We next sought to determine whether, instead of a correlation between endocycle number and CW, there might be a correlation between CW and total DNA content of the PG. In this scenario, the increased number of cells in *fzr* knockdown glands might still lead to the same DNA template number in the gland at CW as wildtype endocycling cells. To this end, we imaged PGs from third instar larvae at the time of CW attainment. We were unable to accurately count cells in the *spok> fzr ^i^ ,Ras ^V12^* line due to their large number. However, for the *spok>, spok>Ras ^V12^ ,* and *spok>fzr ^i^* lines, nuclei of serial z sections from five or six PGs were counted. The results (Figure 1E) show that both the endoreplicating lines, *spok>* and *spok>Ras ^V12^ ,* have approximately 55 PG cells at the time of CW, consistent with prior EM studies which reported a cell number of ~60 (King et al. 1966) cells and Ohhara et al. (2017) who reported ~50 PG cells in wild type *Drosophila* PGs. In contrast, the *spok>fzr ^i^* mitotic PGs had on average 220 or about four-fold more cells than the endoreplicating PGs. This indicates that on average they underwent an additional 2 mitotic cycles compared to the endoreplicating lines. Ohhara et al. showed that in wild type endoreplicating PG cells, CW is attained when the C value transitions from 16 to 32, which is the third endocycle after MES occurs during the first instar to second instar transition. Assuming that the *fzr ^i^* mitotic cells are 2C after division, this means that the DNA template number is approximately 2-fold less than in endoreplicating cells at CW. However, as discussed above, if the *fzr ^i^* cells are tetraploid instead of diploid, then the total DNA content would be the same at CW for both endocycling and the “mitotically” dividing *fzr ^i^* knockdown PG cells. Clearly, this issue will require a more precise determination of C value content in the *spok>fzr ^i^* PG cells to resolve.

Despite the ambiguity concerning total DNA template number in the PG at the time of CW, our data clearly show that endocycles *per se* are not required for normal CW attainment nor adult viability in *Drosophila.* Once sufficient DNA is synthesized in the PG, either by endoreplication resulting in polyploidy or via mitotic division giving rise to increased cell numbers, the ecdysone biosynthetic pathway enzymes can drive ecdysone synthesis adequately to signal the initiation of metamorphosis. We suggest that instead of CW being determined by counting endocycles or even total DNA template number, that it instead represents attainment of a threshold value of ecdysone that is sufficient to activate an EcR-dependent feed-forward loop to further ramp up ecdysone production (Moeller et al. 2013). Many factors influence the biosynthetic capacity of the PG during its maturation, including numerous signaling pathways such as insulin, TOR, TGFβ, HH, EGF (Pan et al. 2021) and Apolpp (Juarez-Carreño et al. 2021), and suggest that there is not likely to be only one pathway that is key to CW determination. Instead, they all play a role in determining when the threshold level of ecdysone production is reached.

Another issue that arises is why the PG switches to endocycles if mitotic cycles can do the job. It could simply be an issue of efficiency. The mitotic cycling flies are developmentally delayed by ~12 hours, and this could be a significant disadvantage in the wild. Another consideration is that the PG normally undergoes programmed cell death during metamorphosis, as do most other polyploid larval tissues. It is possible that the timing of PG death might be perturbed by a mitotic program, and this could once again have subtle, but important, consequences on the fitness of adults in their native environment.

Why are our results different from Ohhara et al.? It is likely to be simply the difference in drivers and the inclusion of *dicer* in the Ohhara studies. We found that if the *phm ^22^ >Gal4* line is used without *dicer* , then approximately 80% of the larvae will pupate with only a slight delay similar to *spok > fzr ^i^* and roughly half of these give rise to viable, normal-sized adults. Perhaps, the extra knockdown that *dicer* supplies uncovers some additional requirement of *fzr* beyond simply control of endocycling, and this requirement was responsible for the observed developmental arrest. Another possibility is that the tissue promiscuity of the *phm* driver coupled with *dicer* leads to knockdown of *fzr* in other endoreplicating tissues, and it is loss of endocycles in another tissue that leads to developmental arrest. However, we note that since feeding 20-hydroxyecdysone to *phm ^22^ >dicer, fzr ^i^* larvaewas able to rescue the developmental delay and pupariation defects of these larvae (Ohhara et al. 2017), this possibility seems less likely. No matter the explanation, our results illustrate the need to use multiple “tissue” specific drivers when characterizing new RNAi phenotypes and to be careful with inclusion of *dicer* in such experiments. In the future, it might also be advisable to confirm novel RNAi-produced phenotypes using Crispr-induced tissue specific loss-of-function studies (Huynh, et al., 2018).

In summary, we propose that the *Drosophila* CW checkpoint likely requires enhanced biosynthetic capacity of the PG through DNA replication, but this need not occur strictly through endoreplication, but can also be largely satisfied through mitotic divisions that increase cell number instead of cell size.

## Methods

Molecular biology: making of *spok-Gal4*

The 1.45 kb fragment of the *spok* enhancer promoter originated as a PCR fragment between the next upstream gene at the time (2009, CG41389 which has since been withdrawn from FlyBase) and the ATG of *spok* . The coordinates are 3R:3321362..3322809 (FB2022_05). The primer pairs used on *w ^1118^* template DNA were *ups spok 01 for* with the sequence 5’ TTCGGTGGAAGGTCCTGACCTTTAG 3’ and *ups spok 02 rev* with the sequence 5’ TTTCAGCCTTAGTAAATAGTTCTCAACATAC 3’. This PCR fragment was cloned into pCRII TOPO (Invitrogen), excised as an EcoRI fragment, and ligated into the EcoRI site of a derivative of pPelican (S. Barolo et al. 2000) where the *β-galactosidase* gene is replaced by *Gal4* (cloned between the BamHI and SpeI sites of pPelican, Lisa Bofenkamp, unpublished) *.*

Fly injections and recombinant screening

The plasmid *pPelican* *spok-Gal4* was injected into *w ^1118^* using P element insertion (BestGene, Chino Hills CA) and generated 26 random insertions. Only insertions on the 2 ^nd^ and 3 ^rd^ chromosomes which were homozygous viable were crossed to *UAS-mCD8-GFP* to assess the strength of expression and possible off-target expression by live imaging whole wandering 3 ^rd^ instar larvae. All lines had about the same level of expression in the PGs and no off-target expression.

Five lines from the 2 ^nd^ chromosome and five from the 3 ^rd^ chromosome were crossed to each other, and 5-8 transheterozygous females were crossed first to *w ^1118^* and subsequently recombinant single males with darker eye color were crossed to Sp/CyO* for recombinants on the 2 ^nd^ chromosome or *TM2/TM6C* for recombinants on the 3 ^rd^ . Recombination events were scored by dark eye color (from 2 copies of mini *white* ) that arose at the same frequency as *white* mutant flies in the cross. Balanced recombinant lines were self-crossed to obtain homozygous stocks.

3 recombinant lines on the 2 ^nd^ or 3 ^rd^ chromosomes, as well as *phm22-Gal4,* were crossed to *UAS-mCD8-GFP.* Ten wandering larvae from each cross were fixed in 4% formaldehyde in PBS Triton (0.2%) for 10 minutes, rinsed in PBS Triton (0.2%), and transferred to 80% glycerol/20% PBS Triton (0.2%). Brain-ring glands were dissected away from the carcass and imaged on a Zeiss Axioplan microscope fitted with a CARV Atto spinning disc attachment. All images were captured under identical conditions with the same number of Z stacks (15 stacks of 2 mm). Z projections made in Fiji (version 2.1.0/1.53c) were measured for integrated density at 2 positions in each PG along with a background subtraction, resulting in 6 measurements for each cross. An average of the integrated density measurements was compared as a ratio of the *phm22-Gal4* measurement. The ratio of *spok-Gal4-16A3,17A3* to *phm22-Gal4* was 1.2 and, based on this number, was chosen for the experiments reported here.

Lastly, *UAS- fzr ^i^* and *UAS-Ras85D ^V12^* were recombined on the 2 ^nd^ chromosome using the same protocol as above. Since the eye color for this recombinant was subtle, we verified the presence of *UAS-Ras85D V12* by PCR of genomic DNA using primers *UAS for* (5’ GAACTCTGAATAGGGAATTGG 3’) and *RasV12 rev* (5’ GTCTCAATGTATGGAATGCCG 3’).

Developmental timing

The method described in Shimell and O’Connor (2022) was used with the exception that 6 vials were used for each cross.

Antibody staining and PG cell number

Either wandering third instar larvae or third instar larvae determined to be near CW by weight measurement were dissected and stained in PB Triton 0.2% with anti-Phospho-histone 3 (PH3) (Cell Signaling Technology, Danvers MA, 1:1000 dilution) and guinea pig anti-Spok (in house generated, 1:1000) primary antibodies and DAPI (20 mg/mL). The secondary antibody for anti-PH3 was goat anti-mouse Alexa Fluor® 555 and for Spok was donkey anti-guinea pig Alexa Fluor® 488. The PGs were stored and dissected in 80% glycerol, 20% PBTriton 0.2% solution and imaged using an LSM 710 microscope (Zeiss).

To count the number of PG cells, serial z stack sections through the PG (1.5 microns) were printed and manually counted for nuclei. Since one nucleus can appear in several sections, the appearance of new nuclei was monitored through the z stacks. Five or 6 individual PGs were analyzed.

Critical Weight measurement

The basic method described in Shimell et al. (2018) was used to determine the critical weights with the following differences. The time between L1 ecdysis and emergence of L3 larvae differed for each cross. That time was 39-40 hours for *spok-Gal4 X UAS-Ras ^V12^ ,* 43-44 hours for *spok-Gal4 X UAS-fzr ^i^* and *spok-Gal4 X UAS-fzr ^i^ ,* *Ras ^V12^* and 41-42 hours for *spok-Gal4 X w ^1118^ .* For *spok>Ras ^V12^* , additional transitions to starvation medium were tested during late L2, just prior to L3 ecdysis, and 1 hour after L3 ecdysis. For the other crosses, transition to starving conditions occurred every 2 hours, as described previously. Feeding-to-starving regimens were carried out to 16-22 hours feeding, or 8-10 hours beyond the point where all starved larvae gave rise to pupae and eclosed adults.

To determine the critical weight, starvation vials that contained a range of larval weights up to the CW were classified into 2 groups that did or did not give rise to pupae. Data from vials was combined up to the time where all starved larvae gave rise to pupae and eclosed adults since, in later vials, the larvae were heavier than critical weight. A line derived by non-linear regression (Prism) of L3 weight gain measurements taken at the feeding-to-starvation transition, was used to interpolate the time at which CW or MVW occurred. Prism recommends using nonlinear regression and not simple linear regression when interpolation of variables is desired.

## Reagents

Fly lines

**Table:** 

*UAS-mCD8-GFP*	BDSC 5137	y ^1^ w*; P{w[+mC]=UAS-mCD8::GFP.L}LL5, P{UAS-mCD8::GFP.L}2	
Sp/CyO*	Lab stock	wg[Sp-1]/CyO S[*]	
*TM2/TM6C*	BDSC 5906	* w ^1118^ /Dp(1;Y)y[+]; TM2/TM6C, Sb ^1^*	
*UAS-RNAi Fzr*	Vienna 25550	*P{w+ UAS-RNAi fzr}.2 (GD)* (gift from D. Fox)	
*UAS-Ras85.V12*	BDSC 64196	w*; P{w[+mC]=UAS-Ras85D.V12}2 RRID:BDSC_64196	
* w ^1118^*	BDSC 5905	* w ^1118^*	
*phm RNAi*	Vienna 6169	* w ^1118^ ; P{GD3430}v6169 *	
*spok RNAi*	(Ono et al. 2006), Lab stock	*spokIR* -2A2	
*dib RNAi*	Vienna 101117	*P{KK106954}VIE-260B*	
*smox RNAi*	BDSC 41670	y ^1^ sc ^*^ v ^1^ sev ^21^ ; P{TRiP.HMS02203}attP40	
* phm ^22^ -Gal4 *	Ono et al. 2006	1.1 kb upstream of *phm* start codon in pPelican Gal4 P element insert on 3 ^rd^ chromosome	
*spok-Gal4 16A3, 17A3*	This work	recombinant of 2 P element inserts of *spok-Gal4* on the 3 ^rd^ chromosome	
*UAS-dicer2 ; phm22-Gal4*	Lab stock	combination stock of VDRC 60008 ( *w[1118]; P{UAS-dicer2, w[+]}* ) and *phm22-Gal4*	

**Software and websites**

FIJI Schindelin, J., Arganda-Carreras, I., Frise, E., Kaynig, V., Longair, M., Pietzsch, T., … Cardona, A. (2012). Fiji: an open-source platform for biological-image analysis. *Nature Methods* , *9* (7), 676–682.  doi:10.1038/nmeth.2019

Prism 9 Version 9.4.0 (June 3, 2022)
